# Pineal Dysgerminoma: A Misleading Clinical Course With Potential Life-Threatening Consequences

**DOI:** 10.7759/cureus.9365

**Published:** 2020-07-23

**Authors:** Alcy R Torres, Carla Salvador, Mauricio D Mora, Wilson Chavez, Javier Romero

**Affiliations:** 1 Pediatrics, Boston University School of Medicine, Boston, USA; 2 Pediatrics Neurology, Boston Medical Center, Boston, USA; 3 Neuroradiology, Massachusetts General Hospital, Boston, USA

**Keywords:** pineal tumor, dysgerminoma, increased intracranial hypertension, tectal syndrome

## Abstract

Pineal dysgerminomas are sporadic pediatric intracranial tumors that usually grow as midline lesions around the third ventricle, most frequently the pineal gland and the pituitary regions of the brain. The severity of symptoms is dependent on the location of the lesion and can present with increased intracranial symptoms. We report a 20-year-old man who presented with new-onset headaches over the past month that would wake him from his sleep at night. The headaches, however, resolved completely one week prior to his first neurological evaluation. A thorough neurological examination was normal. A careful review of the literature does not show a case of a pineal tumor presenting with spontaneous regression of intracranial pressure, and therefore we would like to raise awareness among clinicians about this potential course. A delay in obtaining imaging could have been life-threatening; thus, we recommend a high index of suspicion when patients present with recent symptoms suggesting increased intracranial pressure. Our patient had an excellent outcome two years after his presentation, with appropriate management including drainage of the cerebrospinal fluid, chemotherapy, and radiotherapy.

## Introduction

A germinoma is an intracranial germ cell tumor (iGCT). The incidence of germ cell tumors (GCTs) varies significantly according to geographical location. In North America and Europe, they account for 0.4-3.4% of all pediatric central nervous system (CNS) tumors, whereas Japan and other Asian countries report that CNS GCTs account for up to 11% of all pediatric brain tumors [1]. Peak incidence is during the second decade of life, with a median age at diagnosis of 10-12 years [2]. There is a male prevalence of 1.8:1 in patients with intracranial germinomas and 3:1 in non-germinomatous GCTs (NGGCTs) [1-3].

Most commonly, germinomas grow as midline lesions around the third ventricle, especially the pineal gland and the neurohypophyseal region of the brain. In females, suprasellar tumors are more common (75%), whereas in males, pineal region tumors are more prevalent (70%). Only a small percentage of patients (5%) present with dual intracranial germinoma, which is the involvement of both regions (pineal and suprasellar) [3,4]. Furthermore, 5-10% of iGCTs present with off-midline structures such as the basal ganglia, thalamus, internal capsule, ventricles, and cerebral hemispheres [3,4]. Tumors localized in both midline and off-midline structures are rarely seen [3,4].

The symptoms depend on the location of the mass, which is dependent on the mass's etiology [5]. Pineal tumors usually compress the tectal plate, which can result in obstruction of the Sylvian aqueduct, causing obstructive hydrocephalus [6]. Patients present with signs and symptoms of increased intracranial pressure secondary to mass effect on adjacent structures, such as headache, vomiting, papilledema, lethargy, and somnolence [5,7]. Headaches that awaken the patient in the middle of the night, appear in the morning, and get worse while laying down or with other Valsalva maneuvers, are particularly concerning. Other symptoms suggesting increased intracranial pressure include transient visual obscurations and positional headaches. In the initial evaluation, risk factors for pseudotumor cerebri or idiopathic intracranial hypertension should be considered.

If the pineal lesion produces pressure on the reflex nuclei of the quadrigeminal plate, patients may develop Parinaud's syndrome, known as dorsal midbrain syndrome, which leads to difficulty in upward vertical gaze (sunsetting eyes), blepharospasm, mydriasis, and impaired ocular convergence. Other symptoms associated with pineal GCTs include ataxia, behavioral changes, and a decline in academic performance [8,9]. Spontaneous regression within one week of symptoms in the absence of treatment has not been reported in the literature and its causes are currently not well understood. We present a case of symptomatic germinoma with spontaneous regression of symptoms and discuss possible explanations for this clinical course.

## Case presentation

We report a case of a 20-year-old man with no significant medical history, except for a remote history of a concussion from which he recovered completely. He had a one-month history of new-onset headaches, which had resolved one week before his initial evaluation. His headaches were frequent, woke him up in the middle of the night, were more prominent in the left frontotemporal region, were non-radiating with a pressure sensation, and had a pain intensity of 10/10, especially when bending forward. The headaches were associated with photophobia, nausea, and vomiting. The headaches were not triggered by any specific food or preceded by visual aura. They did not get worse with other Valsalva maneuvers such as coughing. They improved when he lay down and worsened every time he sat up abruptly, sometimes causing dizziness, which he described as lightheadedness. He usually slept in a supine position. There was no history of recent trauma, sinus disease, fever, or toxic exposure. Acetaminophen or ibuprofen had been taken multiple times with no significant improvement. The patient had had a complete resolution of his symptoms one week prior to the time of his neurological evaluation. His family, however, wanted to make sure he did not have a major illness before he returned to college.

General physical examination was unremarkable. Vital signs were normal. He appeared well-developed and well-nourished, and he was in no apparent distress. He had no dysmorphic features or neurocutaneous stigmata. His neck was supple. On neurological examination, he was alert, awake, and attentive. His naming, repetition, and immediate recall were intact. Short-term memory and delayed recall were normal. He had no problem stating the months of the year backward. His backward digit span was normal. He had no right-to-left confusion. He was able to do 100 - 7, five times without a problem. His speech was fluent. He was able to read, write, and copy a geometric figure without difficulty. His cranial nerve examination showed no deficits. His pupils were equal, round, and reactive to light. Extraocular movements were intact. There was no facial asymmetry. Fundus examination showed no papilledema. There was normal facial sensation and normal hearing to rubbing of the fingers on both ears. His palate elevated symmetrically and his tongue was in the midline without fasciculations. He had normal neck strength.

On motor examination, he had normal muscle tone and muscle bulk, and his strength was full and symmetric. His deep tendon reflexes were 2+ throughout. Babinski's sign was negative. He did not present clonus. He did not have sensory deficits. He had no ataxia or dysmetria. Romberg's test was negative. He exhibited no abnormal movements. He was able to do toe-walking, heel walking, and tandem gait. He was able to run with no posturing, and his running was appropriate for age. There were no gait disturbances.

**Figure 1:**
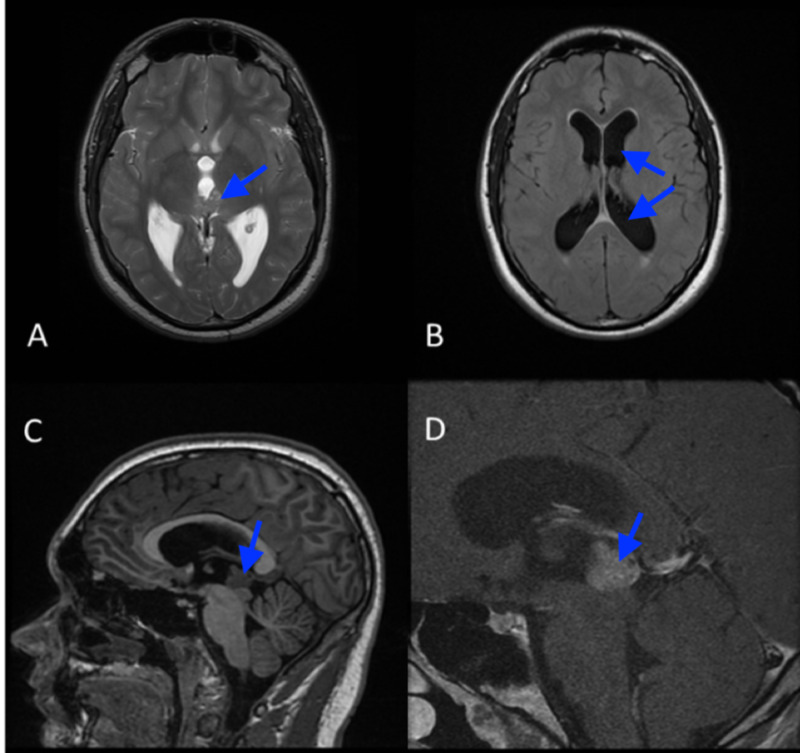
Pre-treatment MRI of the brain. Axial T2-weighted MR image (A) of the brain at the level of the basal ganglia shows a predominantly T2 hypointense mass involving the pineal region (arrow), which causes a mass effect on the cerebral aqueduct and the third ventricle resulting in hydrocephalus, well seen in the axial FLAIR MR image (B). Sagittal T1-weighted MR image (C) shows the lesion is hypointense on T1 and homogeneously enhances on sagittal post-contrast T1-weighted MR image (D). MRI, magnetic resonance imaging; MR, magnetic resonance; FLAIR, fluid-attenuated inversion recovery

Brain magnetic resonance imaging (MRI) with and without contrast was obtained, which showed a predominantly T2 hypointense mass involving the pineal region, causing a mass effect on the cerebral aqueduct and the third ventricle, resulting in obstructive hydrocephalus (Figure 1). The differential diagnoses at that time were GCTs, pineocytomas, pineoblastomas, and gliomas.

Laboratory workup included tumor markers such as HGC (human chorionic gonadotropin) and AFP (alfa-fetoprotein), which were within the normal range. Lumbar puncture (LP) was performed and cytology was negative for malignant cells.

**Figure 2:**
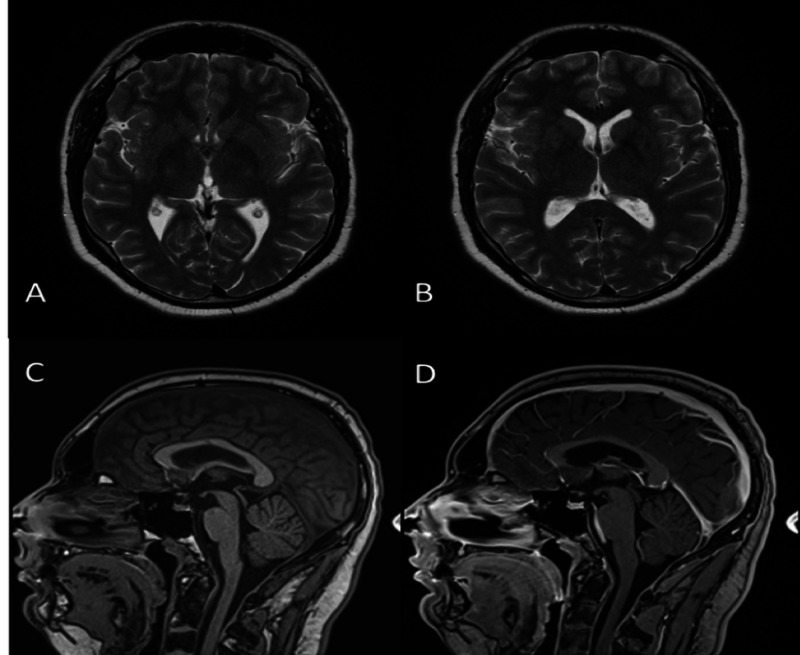
Post-treatment MRI of the brain. Axial T2-weighted MR image (A,B) shows interval resolution of the hydrocephalus post-ventriculostomy. Pre and post-contrast sagittal T1 weight MR image respectively (C, D) showing the absence of the pineal mass post-chemotherapy and radiotherapy. MRI, magnetic resonance imaging; MR, magnetic resonance

A third ventricular fenestration was performed to decompress the hydrocephalus (Figure 2). The biopsy was consistent with a pineal dysgerminoma, and the patient was treated with chemotherapy and radiotherapy (Figure 2).

## Discussion

Space-occupying lesions involving the pineal region produce signs and symptoms related to the mass effect on the adjacent tissues [10] or invasion of the surrounding structures. Due to the size of the third ventricle and the aqueduct of Sylvius, secondary hydrocephalus is a common complication of pineal tumors that are just on top of the entrance of the aqueduct. The most common symptoms of increased intracranial pressure are headache, nausea, and vomiting. The increased pressure eventually results in dorsal midbrain syndrome (Parinaud syndrome) [1,3]. Gait instability and ataxia have also been described; compression of the pituitary infundibulum can lead to diabetes insipidus (most common), hypopituitarism, or optic chiasm compression with diplopia [1,3,4]. The headaches in our patient had features of increased intracranial pressure that spontaneously regressed a week before his neurological evaluation without any treatment and without a clear explanation.

To understand this unusual presentation, we must focus on the importance of the pineal gland anatomy. The pineal gland is a small structure, variable in size of about 5 mm, located in the midline, above the tentorium, and below the splenium of the corpus callosum [11]. It is connected through the pineal stalk to the posterior roof of the third ventricle. Due to its anatomical location, any size changes caused by masses or tumors, such as pineal dysgerminomas, could lead to a mechanical obstruction of the aqueduct of Sylvius, resulting in obstructive hydrocephalus, which does not allow cerebrospinal fluid (CSF) flow through the third to the fourth ventricle, leading to increased intracranial pressure.

We speculate that the improvement in our patient’s symptoms was due to increased pressure inside the third ventricle that was able to open the aqueduct of Sylvius; alternatively, increased compliance of the third ventricle and nearby tissues is possible. Finally, we also contemplated the possibility of a positional change as the tumor increased in size, caused by gravity, but this is not consistent with the patient's initial symptoms. We suspect his symptoms eventually would have returned, but it is not possible to predict how long that would have taken or what would have been his clinical presentation. Most concerning is the possibility of an acute hydrocephalus due to sudden obstruction of the third ventricle, culminating in significant disability or death at the time of the recurrence.

Sato et al. described an 11-year-old boy who presented with polyuria and headache. Head CT and brain MRI revealed tumors in the suprasellar and pineal regions with obstructive hydrocephalus. A repeat brain MRI demonstrated shrinkage of the tumors, resection was deferred, and the patient was discharged with serial MRI for follow-up. The tumor continued to regress for three weeks; however, the patient was readmitted due to a tumor regrowth [12]. The results of the biopsy confirmed that the final histopathologic diagnosis was germinoma. In our patient, however, it is unlikely tumor regression occurred in only one week.

According to Zhang et al., spontaneous regression of iGCTs is a rare phenomenon. Only 10 cases have been reported comprising one female and nine males, with a mean age of 22.1 ± 10.3 years (range:12-43 years). Among those cases, nine were diagnosed as germinoma, and four cases showed regression followed by regrowth [13]. Zhang et al. proposed four hypotheses to explain this regression, including radiation exposure, surgical procedures, effects of steroids, and immunotherapy. No clinical details were available on these patients; however, the progression in our patient occurred before any treatments were started.

The diagnosis of iGCTs is best determined by brain MRI, which is the preferred imaging study due to its high sensitivity. It is the most accurate method to identify the tumor and delineate its relationship to adjacent structures, allowing true pineal masses to be distinguished from parapineal masses [14]. Awa et al. described a study of 93 patients to clarify the imaging features for the differentiation of pineal germinoma and other pineal region tumors classified as germinomas, NGGCTs, pineal parenchymal tumors (PPTs), and miscellaneous tumors of the pineal region. They found certain specific features to differentiate one from another and concluded that a bithalamic extension of the tumor and thick peritumoral edema would be specific to differentiate germinomas from NGGCTs and PPTs [15].

Our patient's brain MRI clearly showed a hypointense mass involving the pineal region; although there was no thick edema, it caused a mass effect on the cerebral aqueduct and third ventricle. At the presentation in the office, even without symptoms, the clinical course prompted imaging and the findings demanded an emergent intervention. The sizeable clinical judgment and intuition prompted the imaging examination and identification of treatable pathology, which improved the clinical prognosis of our patient.

## Conclusions

To our knowledge, our patient is the first-ever reported case in the literature with complete resolution of all clinical symptoms of increased intracranial pressure before any therapies were instituted and normal neurological examination due to a pineal dysgerminoma. While we hypothesize that our patient's symptom resolution may have been due to the increased pressure in the third ventricle or increased compliance of the ventricle and adjacent structures, it is not entirely clear to us why it happened and further research on the dynamics of the CSF might be helpful in the future. Clinicians, however, must be aware of this potentially misleading course and consider appropriate imaging in the setting of new-onset headaches, even if increased intracranial pressure symptoms have resolved, as this might be a life-saving decision.
